# Identification of small-molecule inhibitors of the immune system repressor Regnase-1

**DOI:** 10.1093/narmme/ugag043

**Published:** 2026-10-01

**Authors:** Arthur Millius, Chee-Hong Takahiro Yew, Yuko Misumi, Jian Yu, Daisuke Takaya, Shuhei Miyakawa, Eun-Sol Choi, Hyo-Jun Lee, Tatum Melati Andini, Fahad Khalaf, Kazuhiko Maeda, Kaori Fukuzawa, Genji Kurisu, Shizuo Akira, Soyoung Park, Daron M Standley

**Affiliations:** Department of Genome Informatics, Research Institute for Microbial Diseases (RIMD), The University of Osaka, 565-0871Osaka, Japan; Laboratory for Systems Immunology, Immunology Frontier Research Center (WPI-iFReC), The University of Osaka, 565-0871Osaka, Japan; Laboratory of Host Defense, WPI-iFReC, The University of Osaka, 565-0871Osaka, Japan; Department of Genome Informatics, Research Institute for Microbial Diseases (RIMD), The University of Osaka, 565-0871Osaka, Japan; TechnoPro, Inc. TechnoPro R&D, Company, 650-0047Kobe, Japan; Protein Crystallography Laboratory, Institute for Protein Research, The University of Osaka, 565-0871Osaka, Japan; Graduate School of Pharmaceutical Sciences, The University of Osaka, 565-0871Osaka, Japan; Graduate School of Pharmaceutical Sciences, The University of Osaka, 565-0871Osaka, Japan; Department of Chemistry, Kunsan National University, 54150Gunsan, Republic of Korea; Department of Chemistry, Kunsan National University, 54150Gunsan, Republic of Korea; Department of Genome Informatics, Research Institute for Microbial Diseases (RIMD), The University of Osaka, 565-0871Osaka, Japan; Department of Genome Informatics, Research Institute for Microbial Diseases (RIMD), The University of Osaka, 565-0871Osaka, Japan; Laboratory of Host Defense, WPI-iFReC, The University of Osaka, 565-0871Osaka, Japan; Department of Host Defense, RIMD, The University of Osaka, 565-0871Osaka, Japan; Graduate School of Pharmaceutical Sciences, The University of Osaka, 565-0871Osaka, Japan; Protein Crystallography Laboratory, Institute for Protein Research, The University of Osaka, 565-0871Osaka, Japan; Laboratory of Host Defense, WPI-iFReC, The University of Osaka, 565-0871Osaka, Japan; Department of Host Defense, RIMD, The University of Osaka, 565-0871Osaka, Japan; Center for Advanced Modalities and Drug Delivery System (CAMaD), The University of Osaka, 565-0871Osaka, Japan; Laboratory for Systems Immunology, Immunology Frontier Research Center (WPI-iFReC), The University of Osaka, 565-0871Osaka, Japan; Department of Genome Informatics, Research Institute for Microbial Diseases (RIMD), The University of Osaka, 565-0871Osaka, Japan; Laboratory for Systems Immunology, Immunology Frontier Research Center (WPI-iFReC), The University of Osaka, 565-0871Osaka, Japan

## Abstract

Regnase-1 is an essential RNase that suppresses immune cell activation by targeting the mRNA of pro-inflammatory cytokines such as interleukin 6 (IL-6). Although genetic inhibition of Regnase-1 activity in CD8+ T cells has been shown to enhance antitumor immunity, no molecular inhibitors of Regnase-1 have been reported. By exploiting a library of nucleic acid analogs in conjunction with an RNA displacement assay, we identified multiple candidate competitors of Regnase-1 RNA substrates. Among these, suramin—an approved drug for treating African sleeping sickness—and several of its derivatives displaced Regnase-1 RNA targets with submicromolar affinity, competitively inhibited Regnase-1–mediated degradation of RNA *in vitro*, and enhanced *Il6* mRNA levels and IL-6 production in mouse embryonic fibroblasts. A 2.62 Å crystal structure revealed that suramin bound to the Regnase-1 active site, coordinating with the catalytic Mg^2+^. Quantum chemical calculations further elucidated qualitative interactions between suramin and surrounding residues of Regnase-1. These findings establish suramin as a small-molecule inhibitor of Regnase-1–mediated RNA decay.

## Graphical Abstract

**Graphical Abstract:**
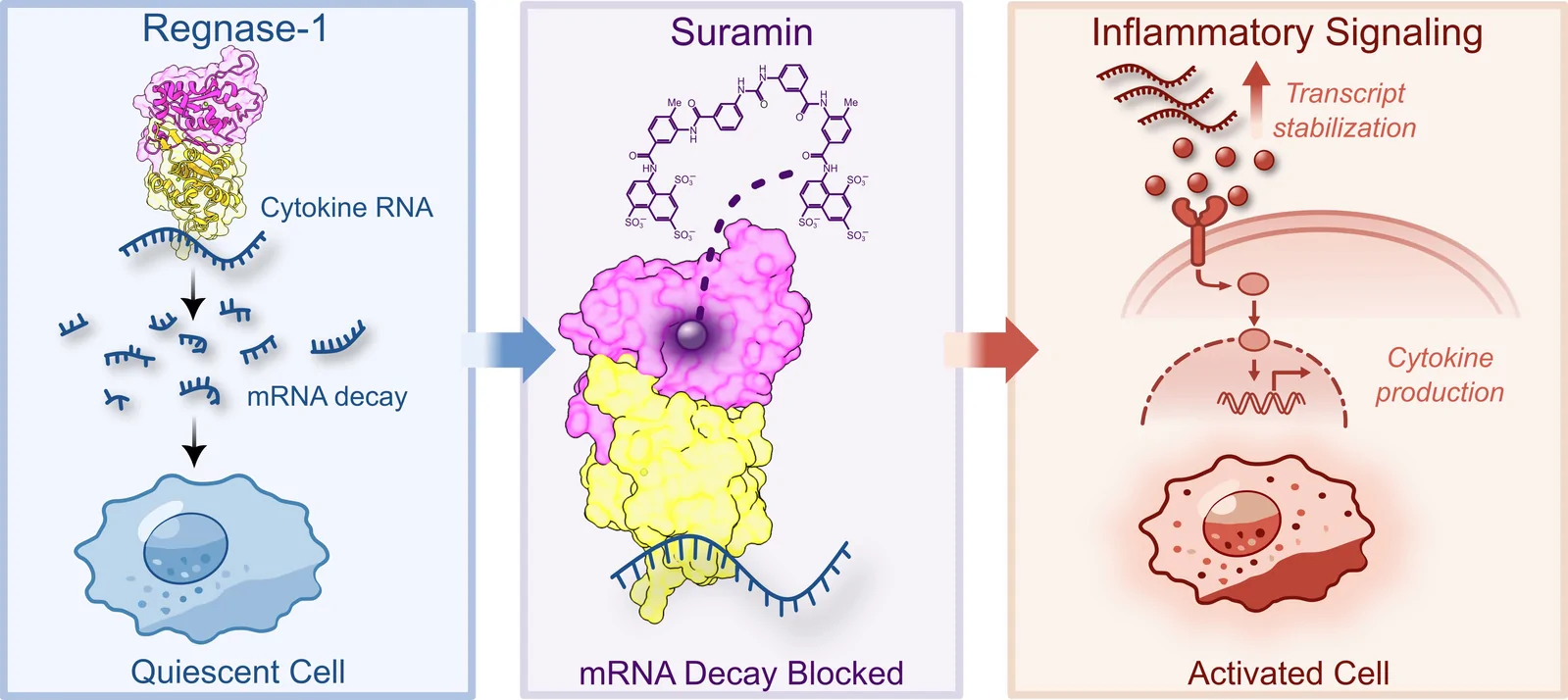

## Introduction

Cancer immunotherapy harnesses the immune system to eliminate tumor cells and produces durable responses in a subset of malignancies [1, 2]. However, tumor heterogeneity, immune evasion, and resistance continue to constrain therapeutic efficacy, underscoring the need for additional molecular targets [3]. Compared with antibody-based agents, small molecule–based immunotherapies offer practical advantages, including lower manufacturing costs, improved oral bioavailability, and enhanced tissue and tumor penetration [4, 5].

Regnase-1 (also known as ZC3H12A or MCPIP1) is an RNase that mediates post-transcriptional regulation of inflammatory responses by degrading the mRNAs of pro-inflammatory cytokines such as IL-2, IL-6, and IL-12B [6–8]. All known Regnase-1 targets contain conserved stem-loop (SL) structures within their 3′-untranslated regions (3′UTRs) [9]. Regnase-1 autoregulates its expression in a negative feedback loop, which can be disrupted by mutating SLs in its own 3′UTR [8, 10]. Loss of Regnase-1 function in mice leads to systemic inflammation, autoantibody production, and early mortality [6]. Conversely, targeted knockout of Regnase-1 in CD8+ T cells confers enhanced antitumor immunity against melanoma and leukemia [11]. Simultaneous disruption of Regnase-1 and Roquin-1 augments immune responses against solid tumors [12], and deletion of Regnase-1 improves CAR-T cell effector function by promoting a proinflammatory tumor microenvironment [13]. Thus, a chemical inhibitor of Regnase-1 activity would be a valuable tool to perturb Regnase-1 function and transiently boost inflammatory responses, but no direct small-molecule inhibitors of Regnase-1 have been reported.

Given the structural features of the Regnase-1 catalytic site, we hypothesized that nucleic acid mimetics could function as competitive inhibitors of Regnase-1–RNA binding. We established a spectral shift RNA-binding assay and a FRET assay based on chemical screening of other RNAses, including IRE1, RNAse L, and RNAse P [14–16], to evaluate Regnase-1 activity. *In vitro* screening of 404 candidate molecules identified suramin—an approved drug for African sleeping sickness—as a potent Regnase-1 inhibitor with submicromolar binding affinity that blocked *Il6* mRNA degradation. X-ray crystallographic analysis revealed that suramin bound directly to the Regnase-1 catalytic site, coordinating with the essential Mg^2+^ ion. Subsequent structure-based exploration of suramin derivatives allowed us to define key structural features that underlie Regnase-1 inhibition. Further analysis of the intermolecular interaction energy between suramin and Regnase-1 by quantum chemical calculations of the complex revealed strong electrostatic and π–π interactions between suramin and residue R283. Together, our findings identify suramin and related compounds as small-molecule Regnase-1 antagonists and suggest a strategy to potentiate immune responses by blocking the post-transcriptional regulation of pro-inflammatory mRNAs.

## Materials and methods

### Reagents, chemicals, and cells

Cyanine5-(Cy5-) and FAM-labeled RNAs (Fasmac or Sigma Genosys) were reconstituted in 10 mM Tris pH 8.0 (Sequences in Supplementary Table S1). Molecular biology reagents and chemicals were purchased from Nacalai or ThermoFisher unless otherwise indicated. Small molecule libraries were obtained from Selleck Chemicals and MedChemExpress and used without additional purification (Supplementary Data S1). Immortalized Regnase-1 KO mouse embryonic fibroblast (MEF) cells have been described previously [17]. MEF cells and 293T cells were cultured in DMEM high glucose (Nacalai), 10% FBS (Gibco), and 1% penicillin/streptomycin (Nacalai).

### Plasmids

Full-length mouse Regnase-1 [6] and a codon-optimized version for *Escherichia coli* expression (IDT) were inserted into pGEX 6p (GE Healthcare). Plasmids expressing domain versions of mouse Regnase-1 have been described [18]. Unstructured and poly proline regions (A97-S133 and P345-P533) in full-length Regnase-1 were replaced by GSSGSSGSS linkers (Regnase-1 NPZC) in the non-codon-optimized construct. PCR mutagenesis was used to create point mutations in the indicated plasmids. Plasmids for luciferase assay have been described [6]. Primers for *Nfkbiz* 3′UTR 100–150 (5′-gacgagctgtacaagTTGTTTCTATGAAACAAACCCATTTAGTTCACTATTATATAGTGGATTATtgatcataatcagcc-3′) were annealed and used for insertion into *Xba*I-digested pGL3 empty plasmid. All constructs were generated using KOD DNA polymerase (Toyobo) and In-Fusion (Takara) and verified by DNA sequencing.

### Expression and purification of Regnase-1

Plasmids were transformed into bacteria Lemo21(DE3) (NEB). Cultures were grown in LB (MP Biomedicals) + 500 µM L-(+)-rhamnose (Nacalai) to an OD600 (0.6–1.3) at 37°C and induced in a final concentration of 0.2 mM isopropyl β-d-1-thiogalactopyranoside at 18°C for 16–20 h. Bacterial cultures were pelleted and stored at −80°C until use. Purification of mouse Regnase-1 protein was similar to that described previously [18]. Briefly, 10 mg lysozyme (Nacalai) per g bacteria cell pellet were lysed in lysis buffer (300 mM NaCl, 20 mM MOPS, HEPES, or Tris pH 7–8, and β-mercaptoethanol), cleared by centrifugation 20 000 × *g* for 30 min, and bound to glutathione sepharose beads (Cytiva). After washing with lysis buffer, proteins were eluted in lysis buffer containing 10 mM glutathione (Nacalai), dialyzed overnight with PreScission protease (Cytiva) in 100 mM NaCl, 20 mM MOPS, Tris, or HEPES pH 7–8, 2 mM MgCl_2_, 1 mM TCEP, and 10% glycerol in 10 kDa molecular weight cut-off dialysis tubing (ThermoScientific). Dialyzed protein was concentrated via cation exchange on a 1 ml SP sepharose gravity column, eluted by a sodium chloride step gradient in dialysis buffer, and dialyzed or size separated by HiLoad 16/60 Superdex 200 or Superdex 75 in gel filtration buffer (100 mM NaCl, 20 HEPES pH 7.0 or Tris pH 8.0, 2 mM MgCl_2_, 1 mM TCEP, and 10% glycerol). Regnase-1 PIN protein for crystallization was size separated in the same buffer lacking glycerol. Fractions were collected and concentrated up to 10 mg/mL with Vivaspin Turbo 4 (Sartorius), aliquoted, and stored at −80°C until use. For Cy5-labeling of Regnase-1 protein, freshly eluted NPZC D141N protein in 20 mM MOPS pH 7.8, 400 mM NaCl, 2 mM MgCl_2_, 1 mM TCEP, and 10% glycerol from SP sepharose cation exchange column was concentrated to 1.2 mg/ml, and 700 µl protein was mixed with 300 µg Cy5 NHS ester (AAT Bioquest) overnight at 4°C. Labeled protein was exchanged into 20 mM MOPS pH 7.8, 100 mM NaCl, 2 mM MgCl_2_, 1 mM TCEP, and 10% glycerol by a PD-10 column (Cytiva), concentrated to 0.5 mg/ml with Amicon Ultra 0.5 ml (Millipore), and frozen at −80°C until use.

### Binding affinity assay of Regnase-1 variants to Cy5-RNAs

The equilibrium dissociation constant (*K*_D_) of the interaction between Regnase-1 variants and Cy5-RNAs was measured using the Monolith X (NanoTemper) as described previously [19]. Cy5-RNA concentration was kept at 40 nM, and Regnase-1 variants were serially 2-fold diluted (16 dilutions) in binding buffer (10 mM NaCl, 20 mM HEPES pH 7, 2 mM MgCl_2_, 1 mM TCEP, and 10% glycerol). To examine the effect of salt on Regnase-1 binding to RNA, affinity assays were performed in binding buffer + 150 mM NaCl or PBS (Nacalai). Samples were loaded into capillaries (NanoTemper or World Precision Instruments), and the spectral shift measurement was performed at 25°C using the Monolith X instrument (NanoTemper) at automated excitation power and medium IR laser power. Ratios of the fluorescence intensities at 670 and 650 nm were used for the analysis of all ligand concentrations using the MO.Control software (v2.5.3, NanoTemper) and plotted in GraphPad Prism.

### RNA displacement assay

A pre-formed Regnase NPZC D141N (200 nM) and 21-nt RNA (40 nM) complex kept at 4°C for <4 h was incubated 1:1 with candidate inhibitors (50 µM) and examined in expert mode using Monolith X (NanoTemper) in binding buffer. RNA binding was normalized to that of dimethyl sulfoxide (DMSO) vehicle (100% bound). Candidate inhibitors were serially 2-fold diluted in binding buffer in ternary binding mode and incubated 1:1 with a pre-formed complex of Regnase NPZC D141N (200 nM), Regnase NPZC (200 nM), or PIN (25 µM) and 21-nt RNA (40 nM) to determine IC_50_.

### Gel-based cleavage assay

Regnase-1 NPZC (400 nM), PIN-ZF (600 nM), or PIN (20 µM) and 200 nM Cy5-labeled *Il6* 3′UTR 84–103 RNA or 200 nM Cy5-labeled or Fam-labeled 21-nt RNA were incubated in 100 mM NaCl, 20 mM Tris pH 7, 2 mM MgCl_2_, 1 mM TCEP at 37°C for 30 min in the absence or presence of the indicated inhibitor (60 µM) or the indicated concentration of suramin. The reaction was stopped with 2× denaturing loading buffer containing 98% (vol/vol) formamide, 10 mM EDTA, and 300 µg/ml xylene cyanol; and boiled at 95°C for 5 min. RNAs were electrophoresed at room temperature in 15% TBE-Urea gels (ThermoFisher) for 45 min at 180 V in 1× TBE and analyzed (Fusion FX, Vilber).

### FRET-based RNAse activity assay

Dual-labeled *Nfkbiz* 3′UTR 100–118 RNA (5′FAM-GUUGUUUCUAUGAAACAAA-3′BHQ1) was purchased from Fasmac. The RNase activity assay was carried out in binding buffer containing 0.05% v/v TritonX-100, at a total reaction volume of 20 μl in a 384-well transparent-bottom, black plate (PerkinElmer). For Michaelis–Menten kinetic studies, a final concentration of 50 nM Regnase-1 NPZC protein and 2-fold serial dilution of 4 µM to 31.25 nM *Nfkbiz* RNA was used. Suramin and Regnase-1 NPZC protein were incubated for 30 min at room temperature before the substrate was added to the reaction. The plate was then kinetically read over three hours at room temperature using a Varioskan plate reader. The measurement was carried out using the fluorescence top reading mode at the excitation wavelength of 485 nm and the emission wavelength of 520 nm with a 12 nm excitation and emission bandwidth. The linear part of the reaction curve was fitted with linear regression to obtain the slope, which reflects the reaction rate, and values were plotted in GraphPad Prism 10.

### Luciferase assay

Luciferase assays were performed and analyzed as described [10]. Briefly, ∼40 000 HEK293T cells were plated in 100 µl culture media of each well of a 96-well plate (Greiner Bio-One). The following day, cells were transfected with 25 ng expression (e.g. pCMV *Regnase-1*), 25 ng reporter (e.g. pGL3 *Il6* 3′UTR 1–400), and 2.5 ng normalization (pRL SV40) plasmids using 0.3 µl of 1 mg/mL PEI MAX (Polysciences) in 25 µl Opti-MEM (Gibco) per well. The day after transfection, cells were treated by the indicated concentration of suramin or vehicle control using 2 µl of 1 mg/mL PEI MAX in 10 µl Opti-MEM per well for 24 h and measured using the Dual-Luciferase Reporter System (Promega) according to the manufacturer’s instructions.

### Quantitative polymerase chain reaction

Approximately 200 000 wild-type or Regnase-1 KO MEF cells were plated in each well of a six-well plate (Corning). The following day, culture media was replaced with 1 ml media containing 500 ng/ml phorbol 12-myristate 13-acetate (PMA, Funakoshi) and 500 ng/ml ionomycin (Sigma) and the indicated concentrations of suramin complexed with 20 µl of 1 mg/ml PEI MAX per well. After 24 h, total RNA was isolated with Trizol (ThermoFisher) according to the manufacturer’s instructions, and digested with DNAseI (NEB) for 30 min at 37°C. Quantitative polymerase chain reaction (qPCR) was performed and analyzed as described [10]. Briefly, cDNA was synthesized with the Revertra Ace Kit (Toyobo), amplified by Thunderbird Probe Onestep qRT-PCR Kit (Toyobo) using *Il6* and *β-actin* TaqMan probes (Applied Biosystems), detected with a ViiA-7 Real-Time PCR, and analyzed with Design and Analysis 2.8.0 (Applied Biosystems).

### ELISA

Approximately 10 000 wild-type or Regnase-1 KO MEF cells were plated in 100 µl culture media of each well of a 96-well plate (Corning). The following day, cells were treated by the indicated concentrations of suramin or vehicle using 2 µl of 1 mg/ml PEI MAX in 10 µl of Opti-MEM per well and then stimulated with 500 ng/ml PMA and 500 ng/ml ionomycin, 500 ng/ml lipopolysaccharide, 50 ng/ml IL-1β, or 50 ng/ml TNF-α and IL-17 overnight. IL-6 concentrations in culture supernatants were measured using an ELISA kit (R&D systems) according to the manufacturer’s instructions.

### X-ray crystallography

Crystallization of mouse Regnase-1 PIN protein was performed essentially as described [18]. Briefly, drops (1 µl) of 6–12 mg/ml Regnase-1 PIN DDNN (residues 134–305; D226N, D244N) in 25 mM NaCl, 20 mM Tris pH 8, 2 mM MgCl_2_, and 1 mM TCEP were mixed with reservoir solution consisting of 0.9 M NaH_2_PO_4_, 0.4 M K_2_HPO_4_, and 100 mM sodium citrate pH 5.5, and crystals were formed by sitting drop vapor diffusion at 20°C after 24–48 h. Crystals were soaked and cryoprotected in a step gradient of reservoir solution + 5% glycerol and 250 µM suramin for 5 min each to a final concentration of 20% glycerol and 1 mM suramin and left for 1 h or overnight before freezing in liquid nitrogen. Diffraction data were collected at SPring-8 beamline BL44XU, and images were processed with xds [20]. An initial model was built based on PDB ID: 5h9v [18] in phenix [21, 22]. Suramin (PDB ID: SVR) was added using Phenix.ligandfit, and the structure was iteratively refined using Phenix.refine and coot [23]. Data collection and refinement parameters are summarized in Supplementary Table S2.

### Fragment molecular orbital calculation

The fragment molecular orbital method [24, 25] is an approximate quantum chemical calculation technique for large biomolecules such as proteins and nucleic acids, based on a partition approximation. By defining fragments at the amino acid residue or compound unit level, it enables the characterization of the interaction type and energies between fragments for a given structure. This method facilitates the evaluation of intermolecular interactions and generates ideas for molecular design, making it widely utilized in various applications related to structure-based drug discovery [25–27]. Using Molecular Operating Environment (MOE) [28], the co-crystal structure of Regnase-1 bound to suramin was modified by replacing N226 and N244 with wild-type aspartic acids, then subjected to hydrogen addition and energy minimization. The theoretical level of the FMO calculations were performed at FMO2–MP2 [29] with the 6–31G* basis set using ABINIT-MP program [25]. The inter-fragment interaction energies (IFIE) obtained from the FMO calculations were further decomposed by pair interaction energy decomposition analysis (PIEDA) into four energy components: electrostatic (ES), exchange repulsion (EX), charge transfer with mixed terms (CT), and dispersion interaction (DI) [26]. Hydrogen bonds are primarily detected by stabilization energies in the ES and CT components, CH/π and π−π interactions are mainly detected by stabilization in the DI component [30, 31]. All structure files and a set of input/output files used for FMO calculations are available at the FMODB [32]; FMODBID is 1818Z for the wild-type structure of Regnase-1 with suramin. Simple data analysis can be performed using the FMODB web interface, and detailed analysis can be performed using the BioStation Viewer software (https://fmodd.jp/biostationviewer-dl/).

### Structure-based virtual screening

For structure-based virtual screening (SBVS), the Regnase-1–suramin crystal structure was employed as the receptor following suramin removal. Regnase-1 and all ligand structures (59 353 compounds) were processed by adding hydrogens with the Protonate3D module and performing energy minimization in MOE [28]. A total of 84 686 conformations (MW ≤ 1000) from The University of Osaka Compound Library were first docked with AutoDock Vina (version 1.2.6) [33, 34] using a search box centered at the centroid of suramin (box size: 20 × 20 × 20 Å; exhaustiveness: 32; num_modes: 100). The top 8500 compounds ranked by Vina score were subsequently subjected to redocking using MOE Dock with the following parameters: Site defined as Pocket Atoms, Placement using Triangle Matcher (100 poses), Refinement with a rigid receptor, Amber10:EHT force field, and scoring based on GBVI/WSA dG (30 poses retained). The MOE Dock results were screened to retain compounds predicted to coordinate Mg, defined by the presence of an atom within 3 Å of the Mg atom, and the top 100 were ranked by the docking score by MOE.

### Design of S0802 and synthesis

To a solution of 5-nitroisophthalic acid (422 mg, 2.0 mmol) in CH_2_Cl_2_ (5 ml) was added oxalyl chloride (0.38 ml, 2.2 eq.), two drops of dimethylformamide (DMF) were then added dropwise. The resulting mixture was stirred at 25°C for 3 h and then concentrated *in vacuo* to afford the corresponding acyl chloride. Separately, 2-amino-5-sulfobenzoic acid (956 mg, 2.2 eq.) was dissolved in H_2_O (8 ml) and the pH was adjusted to 3.5 by addition of a 2 M aqueous solution of Na_2_CO_3_. To the stirred aqueous solution was added dropwise a solution of the acyl chloride in toluene (1 ml). The reaction mixture was kept at a constant pH of 3.5 by addition of a 2 M aqueous solution of Na_2_CO_3_ and stirred overnight at 25°C. After separation of the toluene phase, the aqueous phase was adjusted to pH 2.0 with 1 M aqueous solution of HCl and extracted with Et_2_O (30 ml). The aqueous phase was then neutralized to pH 7.0 by addition aqueous solution of Na_2_CO_3_ and concentrated *in vacuo*. The crude product was purified by recrystallization from methanol. The recrystallized solid was subsequently dissolved in H_2_O, and the solution was basified to pH 10.0 by addition of 2 M aqueous solution of Na_2_CO_3_, followed by concentrated *in vacuo*. The residue was further purified by recrystallization from methanol to afford sodium salt of S0802 as a yellow solid (770 mg, 59% yield) and characterized by NMR (Supplementary Fig. S8).

## Results and discussion

### Engineered Regnase-1 and selection of target RNAs for chemical screening

To enable biochemical screening for RNA displacement, we generated a modified Regnase-1 construct containing the N-terminal (NTD), PilT N-terminal like (PIN), zinc finger (ZF), and C-terminal (CTD) domains (termed NPZC) that improved protein yield while retaining catalytic activity (Supplementary Fig. S1). The NPZC construct contained all structured domains, was expressed at much higher yield than codon-optimized full-length Regnase-1 (Supplementary Fig. S1A–C), and cleaved *Il6* RNA with similar efficiency to full-length Regnase-1 *in vitro* (Supplementary Fig. S1D and E) and in cells (Supplementary Fig. S1F). To identify preferential RNA targets of Regnase-1 for subsequent displacement assays, candidate RNAs (Supplementary Table S1) were evaluated in a spectral shift assay. A 21-nt synthetic RNA discovered by HTR-SELEX [35] displayed the strongest binding (Fig. 1A and B), whereas other known Regnase-1 targets in cells, such as *Il6* 3′UTR 84–103, *Nfkbiz* 3′UTR 100–118, and *Regnase-1* 3′UTR 193–213 [6, 8, 36], had much lower affinity for Regnase-1. Furthermore, binding of the 21-nt RNA to Regnase-1 was substantially weakened by physiological concentrations of salt (Fig. 1C). Thus, Regnase-1 binding to RNA is primarily driven by electrostatic interactions, which suggests that in cells other proteins are likely necessary to help bind and deliver RNA to Regnase-1 for cleavage. We selected the 21-nt RNA for displacement assays in low salt conditions in order to discover chemical inhibitors that might perturb Regnase-1-RNA binding (Fig. 1D).

**Figure 1.**
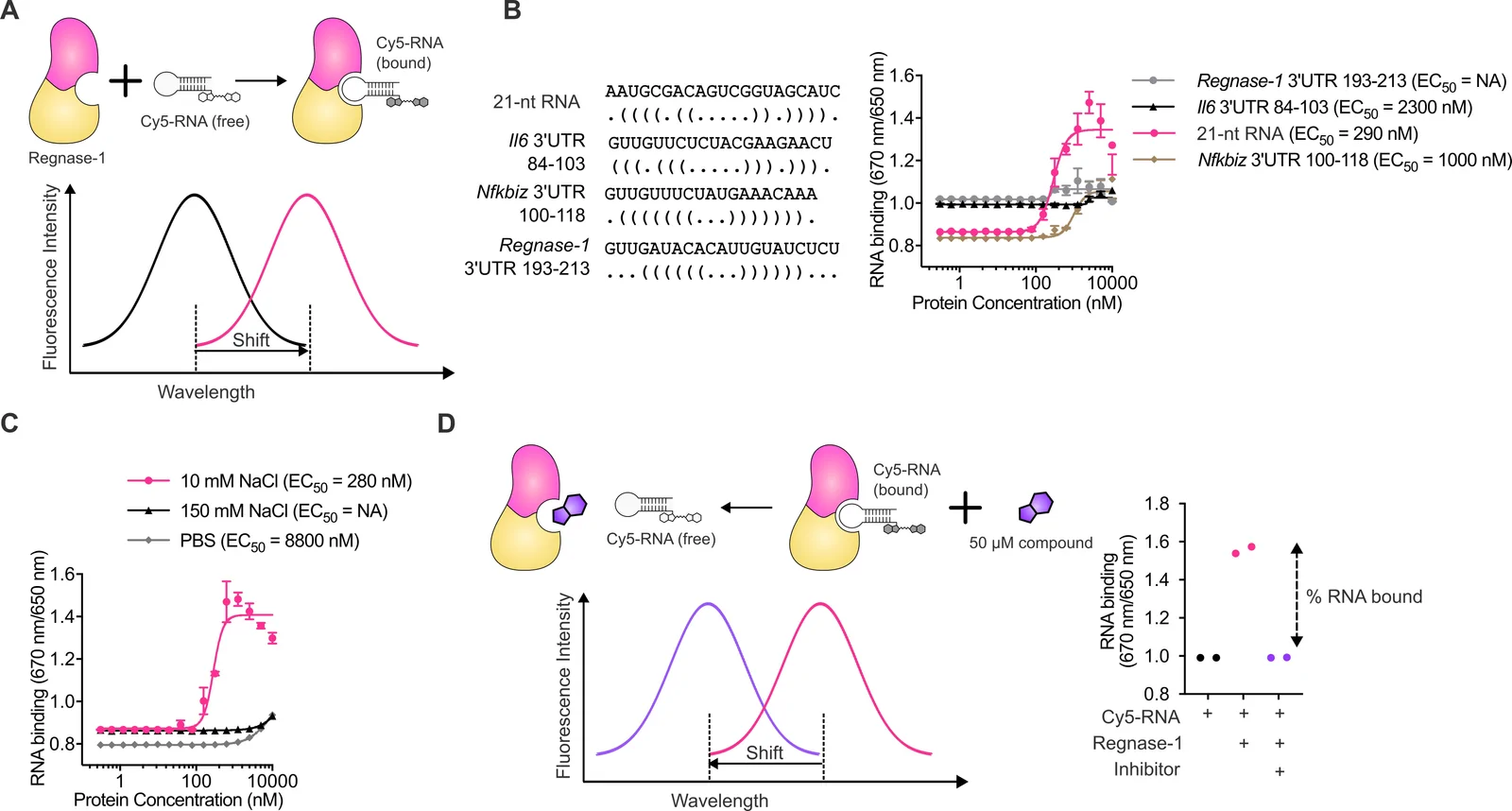
Selection and characterization of Regnase-1-binding RNAs for chemical screening. (**A**) Principle of the Monolith spectral shift assay. (**B**) Left, candidate RNAs and their predicted secondary structure. Right, affinity assays of the indicated Cy5-labeled RNA with Regnase-1 NPZC D141N. (**C**) Affinity assays of 21-nt RNA with the Regnase-1 NPZC D141N in 10 mM NaCl binding buffer (pink), 150 mM NaCl binding buffer (black), or PBS (gray). Data are mean ± s.e.m. in (B) and (C) (*n* = 3). (**D**) Left, principle of chemical inhibition of Regnase-1-RNA binding in the spectral shift assay. Right, illustration of a spectral shift assay showing 100% displacement of bound RNA.

### Small-molecule screen identifies RNA competitors of Regnase-1

Given that Regnase-1 is an RNA-binding protein with endoribonuclease activity [37], we constructed a focused library primarily composed of nucleoside and nucleotide derivatives. During initial screening, several compounds previously identified as P2Y12 and P2X3 receptor ligands were found to disrupt RNA binding likely because these compounds tend to be heavily polyanionic or aromatic, which mimics nucleic acids and might have cross reactivity [38]. Based on these findings, we expanded the library to incorporate additional compounds known to target the P2Y12 and P2X3 receptors, ultimately assembling a collection of 404 compounds (Supplementary Data S1).

We screened compounds for ones that disrupt the interaction between Regnase-1 and its target RNA, quantifying the fraction of Cy5-labeled RNA bound to Regnase-1 NPZC relative to free RNA (Fig. 2A). Suramin, salvianolic acid B, and A317491 reproducibly emerged as the most potent competitors of the target RNA (Fig. 2B and C, and Supplementary Fig. S2). These compounds share a common pharmacophore consisting of hydrophobic motifs formed by benzene or naphthalene rings, in combination with polyanionic sulfate or carboxylate groups capable of coordinating cationic metal ions. These distinctive chemical features facilitate hydrogen bond formation, charge interactions, and hydrophobic packing with amino acid residues in the PIN domain of Regnase-1. Binding assays demonstrated a direct interaction exclusively between suramin and Cy5-labeled Regnase-1 NPZC (Supplementary Fig. S2C and D). Suramin inhibited RNA binding with an IC_50_ of 48 nM (Fig. 2D), the lowest among all library compounds screened. A gel-based cleavage assay demonstrated that suramin, but not A317491 or salvianolic acid B, effectively blocked Regnase-1–mediated RNA degradation (Supplementary Fig. S2E) with IC_50_ = 1300 nM (Fig. 2E). The roughly 25-fold difference in suramin efficacy between binding and cleavage might reflect differences in assay design or in differences in the ability between wild-type and D141N protein to bind RNA. Remarkably, we could observe similar binding affinities of wild-type Regnase-1 NPZC to the 21-nt RNA (EC_50_ = 390 nM) and *Nfkbiz* 3′UTR 100–118 (EC_50_ = 1780 nM) in the short period before RNA cleavage (Supplementary Fig. S2F). However, suramin was less able to disrupt binding of wild-type Regnase-1 NPZC to 21-nt RNA (IC_50_ = 760 nM, Supplementary Fig. S2G), similar to the results of the gel-based cleavage assay. In the absence of Regnase-1, suramin did not bind or cleave RNA, and suramin blocked cleavage of RNA by PIN domain alone in the absence of other domains of Regnase-1 (Supplementary Fig. S3A and B). Furthermore, suramin had little effect on RNAse A (Supplementary Fig. S3C), which suggests that suramin does not inhibit ribonucleases indiscriminately.

**Figure 2.**
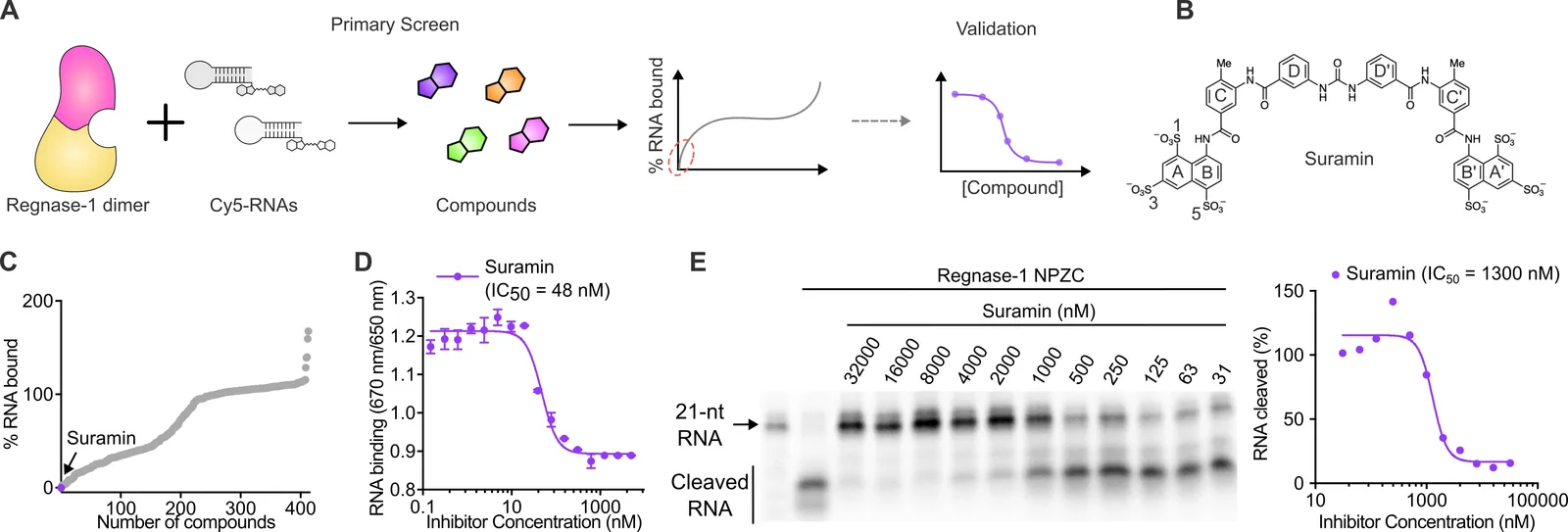
A chemical screen identifies small-molecule inhibitors of Regnase-1 (**A**) Screening workflow. (**B**) Chemical structure of suramin. (**C**) Summary of the primary screen. Normalized values of the fraction of Cy5-labeled 21-nt RNA (gray) bound to Regnase-1 NPZC D141N treated with compound versus unbound RNA, where DMSO negative control treatment is 100% RNA bound. Arrow indicates % RNA bound in the presence of suramin (purple). (**D**) IC_50_ of suramin for the Regnase-1-RNA complex (*n* = 6, mean ± s.e.m). (**E**) *In vitro* cleavage assay of Regnase-1 NPZC to FAM-labeled 21-nt RNA with different concentrations of suramin. The fluorescence intensity of the cleaved RNA was quantified (right).

### Suramin is a competitive inhibitor of Regnase-1 RNase activity

To understand the kinetics of Regnase-1-mediated RNA cleavage, we developed a fluorescence resonance energy transfer (FRET)-based assay using a dual FAM- and BHQ-labeled *Nfkbiz* 3′UTR 100–118 RNA (Fig. 3A). The stem-loop structure of the RNA brings the BHQ quencher in close proximity to the FAM until enzymatic cleavage. Increasing the amount of Regnase-1 NPZC showed that fluorescence intensity depended on protein concentration (Fig. 3B) and revealed ideal enzyme concentrations (<500 nM) to ensure a linear initial velocity (Supplementary Fig. S4A–C). To investigate the inhibition mechanism of suramin, a Michaelis–Menten analysis was performed over a range of RNA substrate concentrations (Fig. 3C), which revealed that the *K*_m_ was 19 nM and that the *V*_max_ was 0.0187 FI/min (fluorescence intensity/minute) in the absence of suramin. Incubation with 500 and 1000 nM suramin increased the *K*_m_ to 74 and 581 nM, respectively, in a concentration-dependent manner (Fig. 3D and Supplementary Fig. S4C). In contrast, the calculated *V*_max_ remained relatively unchanged as the *V*_max_ for 500 and 1000 nM suramin were 0.0144 and 0.0165 FI/min, respectively. A classic Lineweaver–Burk double-reciprocal plot showed a similar *y*-intercept (1/*V*_max_) and an increase in the apparent slope (*K*_m_/*V*_max_) with an increase in suramin (Supplementary Fig. S4D). Thus, the overall constant *V*_max_ and concentration-dependent *K*_m_ suggested a competitive inhibition mechanism.

**Figure 3.**
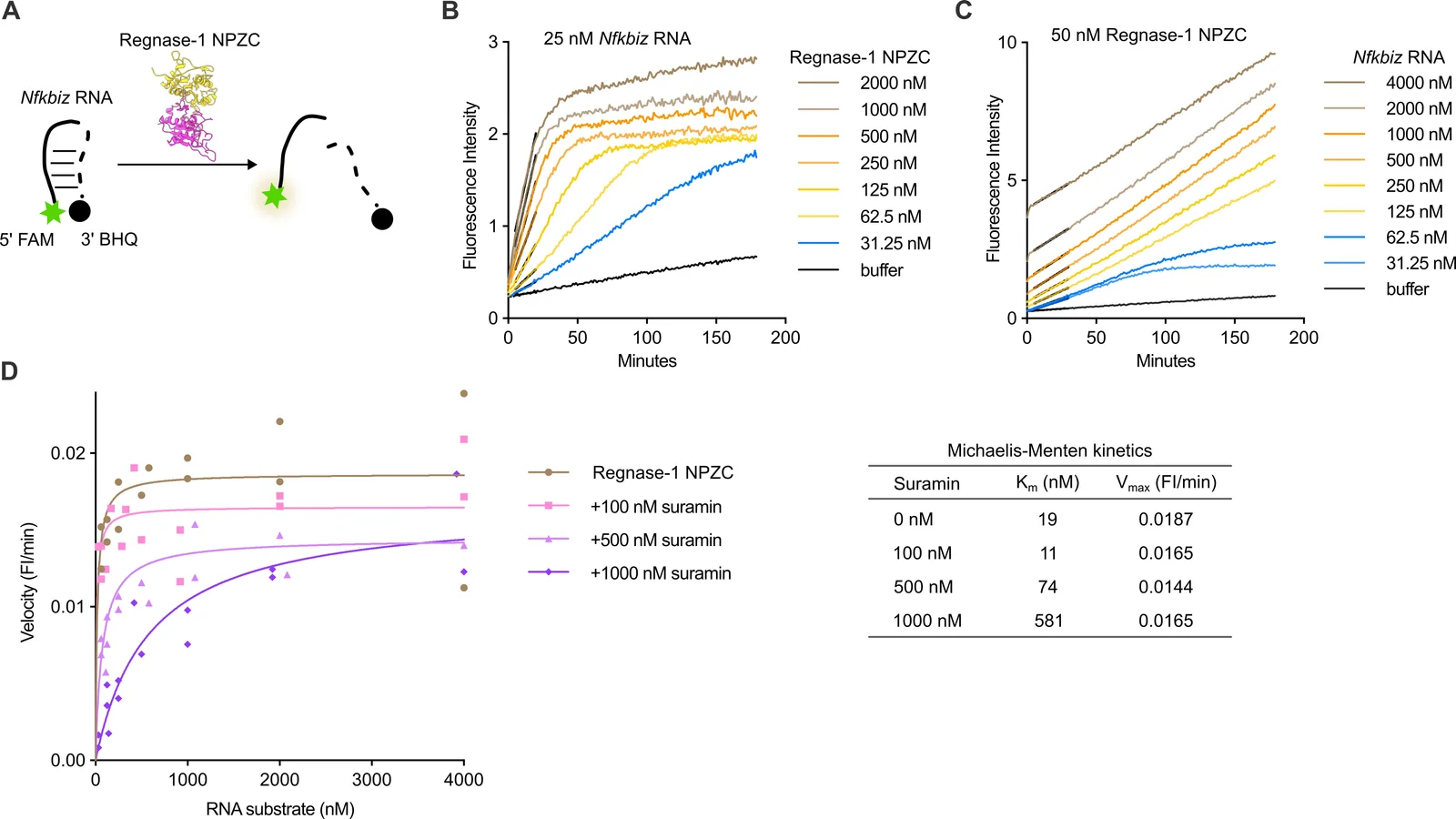
Suramin is a competitive inhibitor of Regnase-1. (**A**) FRET assay to monitor Regnase-1 RNAse activity. (**B**) Titration of Regnase-1 NPZC protein and (**C**) *Nfkbiz* 3′UTR 100–118 RNA in the FRET-based cleavage assay. Short black lines indicate linear regression used to calculate the slope in the linear part of the reaction curve. Data plotted are mean (*n* = 2). (**D**) Michaelis–Menten kinetic study showed that suramin is a competitive inhibitor of *Nfkbiz* 3′UTR 100–118 RNA (*n* = 2).

### Suramin inhibits Regnase-1 in cells

Although Regnase-1 exhibits sequence and structural specificity in RNA cleavage in cells [36], it degrades most RNAs *in vitro* regardless of sequence or structure [17, 39–41]. Because suramin is known to bind proteins with positively charged electrostatic surfaces [42–46], its inhibitory effect in cells is likely pleiotropic. Nevertheless, we examined the effect of suramin in HEK293T cells and found that expression of an *Il6* 3′UTR luciferase reporter increased in a suramin concentration-dependent manner (Fig. 4A). In addition, suramin increased IL-6 production and *Il6* mRNA levels in stimulated MEFs to an extent comparable to Regnase-1 knockout MEFs (Fig. 4B and C). To determine the pleiotropic effects of suramin in the absence of Regnase-1, we treated Regnase-1 knockout MEFs with a variety of chemical stimuli and measured IL-6 levels by ELISA (Fig. 4D). As expected, suramin decreased IL-6 levels in response to lipopolysaccharide (LPS), IL-1β, and TNF-α plus IL-17, which reflects a known role for suramin in inhibiting signaling pathways downstream of LPS activation and in inhibiting the interaction of IL-1β and TNF-α with their receptors [47–50]. In contrast, suramin had less effect on IL-6 levels in response to PMA plus ionomycin stimulation in Regnase-1 knockout MEFs (Fig. 4D), which suggests that the effect of suramin on IL-6 levels in this pathway is through Regnase-1. Together, these data suggest that suramin can functionally inhibit Regnase-1 to increase IL-6 levels in cells in response to PMA and ionomycin stimulation.

**Figure 4.**
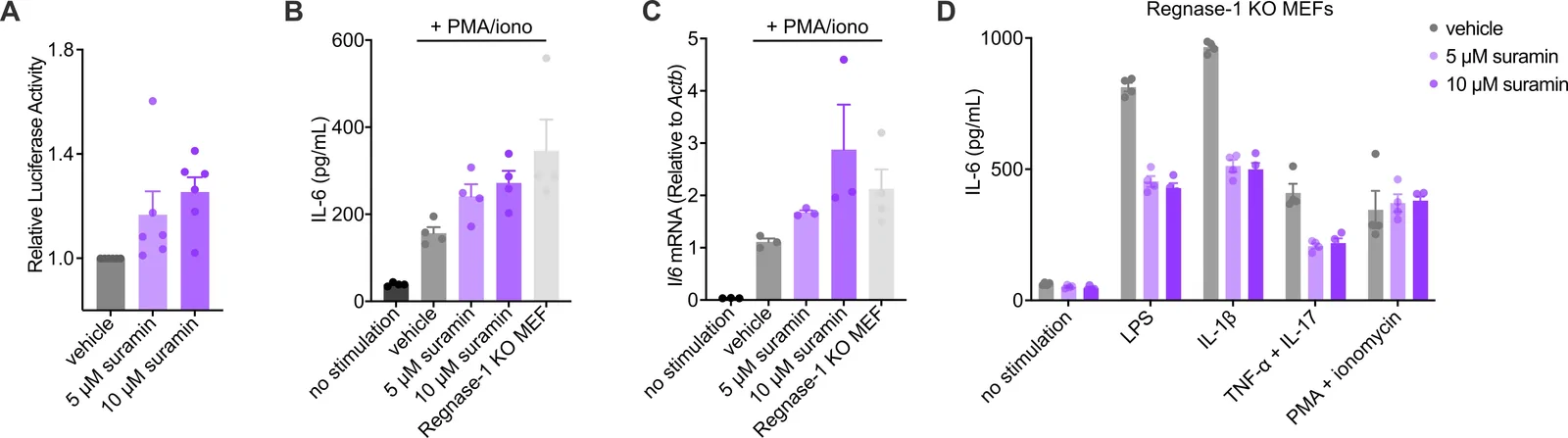
Suramin increases IL-6 levels in cells in response to PMA and ionomycin treatment. (**A**) Luciferase assay of HEK293T cells 48 h after transfection with *Il6* 3′UTR 1–400 Firefly luciferase reporter plasmid normalized to a *Renilla* control plasmid treated for 24 h with suramin. Values are normalized to treatment with vehicle (*n* = 3). (**B**) Production of IL-6 cytokine measured by ELISA (*n* = 4) and (**C**) *Il6* mRNA levels measured by qPCR (*n* = 3) in wild-type MEF cells treated with suramin or vehicle in response to PMA + ionomycin after 24 h compared to Regnase-1 KO MEFs. (**D**) Production of IL-6 cytokine in Regnase-1 KO MEFs treated with suramin or vehicle in response to the indicated stimuli measured by ELISA (*n* = 4). All data are mean ± s.e.m.

### Structural basis of suramin inhibition of Regnase-1

Regnase-1 contains a single catalytic site within the PIN domain, positioned adjacent to an RNA-binding CCCH ZF domain. The NTD and CTD, whose biochemical functions remain unclear, are connected to the PIN and ZF domains by flexible linker regions. This intrinsic flexibility has hindered structural characterization of both the full-length protein and the NPZC construct. In contrast, the isolated PIN domain crystallizes as a dimer, and residues at the interface have been shown to be required for catalytic activity [18, 35, 51]. The catalytic center comprises four aspartate residues that coordinate an essential Mg^2+^ ion.

Wild-type and endonuclease-deficient Regnase-1 PIN domain (D226N, D244N) crystals were soaked with a 3-fold molar excess of suramin (Materials and methods). The wild-type crystals diffracted with low resolution and were discarded, but the mutant PIN crystals (space group P3_2_21) diffracted to a resolution of 2.62 Å (Fig. 5; PDB ID: 23KX, Supplementary Table S2). The PIN domain formed a head-to-tail dimer, and superposition with the apo-PIN structure (PDB ID: 3v33) yielded an overall root mean squared deviation (r.m.s.d.) of 0.594 Å (Supplementary Fig. S5A). Suramin was bound with 1:1 stoichiometry within the active site of each PIN protomer, with clear electron density for its terminal sulfonate groups and coordination with the catalytic Mg^2+^ ion (Supplementary Fig. S5B). Interestingly, residues R283 and H284 from the opposing protomer interacted with the opposite end of suramin via hydrogen bonding and π–π stacking (Fig. 6A–C; SupplementaryTable S3). Given the high similarity between Regnase family members in the PIN domain, it is highly plausible that they would also be affected by suramin [52]. Because the Mg^2+^ ion is retained in the structure of the endonuclease-deficient PIN structure, we think suramin docks to wild-type PIN in a similar manner, but future high-resolution structural studies are needed to answer this question definitively.

**Figure 5.**
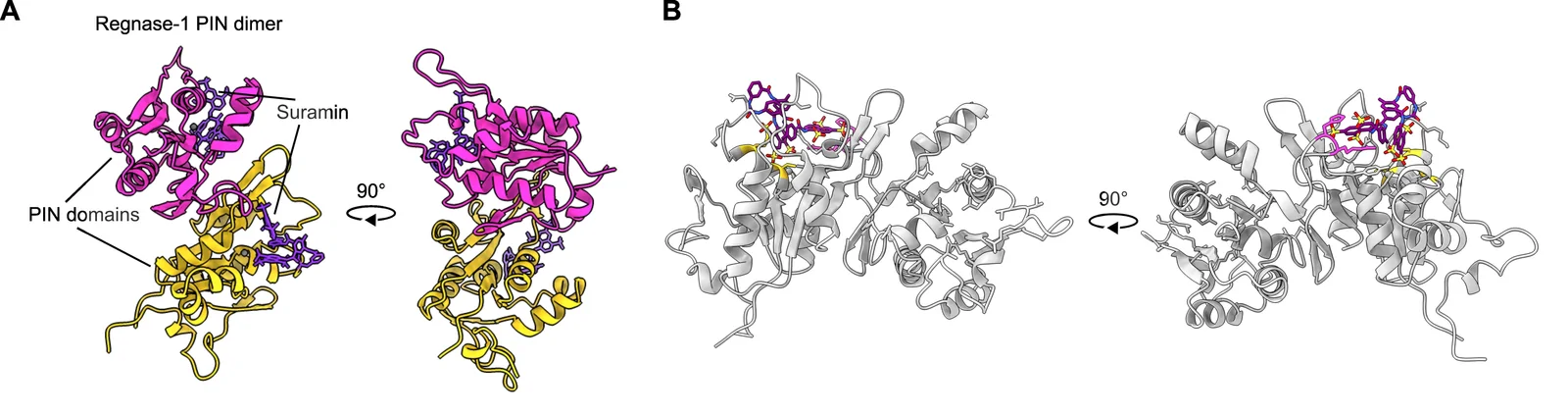
Overall structure of the Regnase PIN–suramin complex. (**A**) Two views of the model of Regnase-1 PIN–suramin complex (PDB ID: 23KX). The PIN dimer consists of one PIN monomer bound in a head-to-tail orientation to the other PIN monomer. A suramin molecule binds to a Mg^2+^ ion at the active site of each PIN monomer. (**B**) Interaction of suramin with residues that contact the PIN domain.

**Figure 6.**
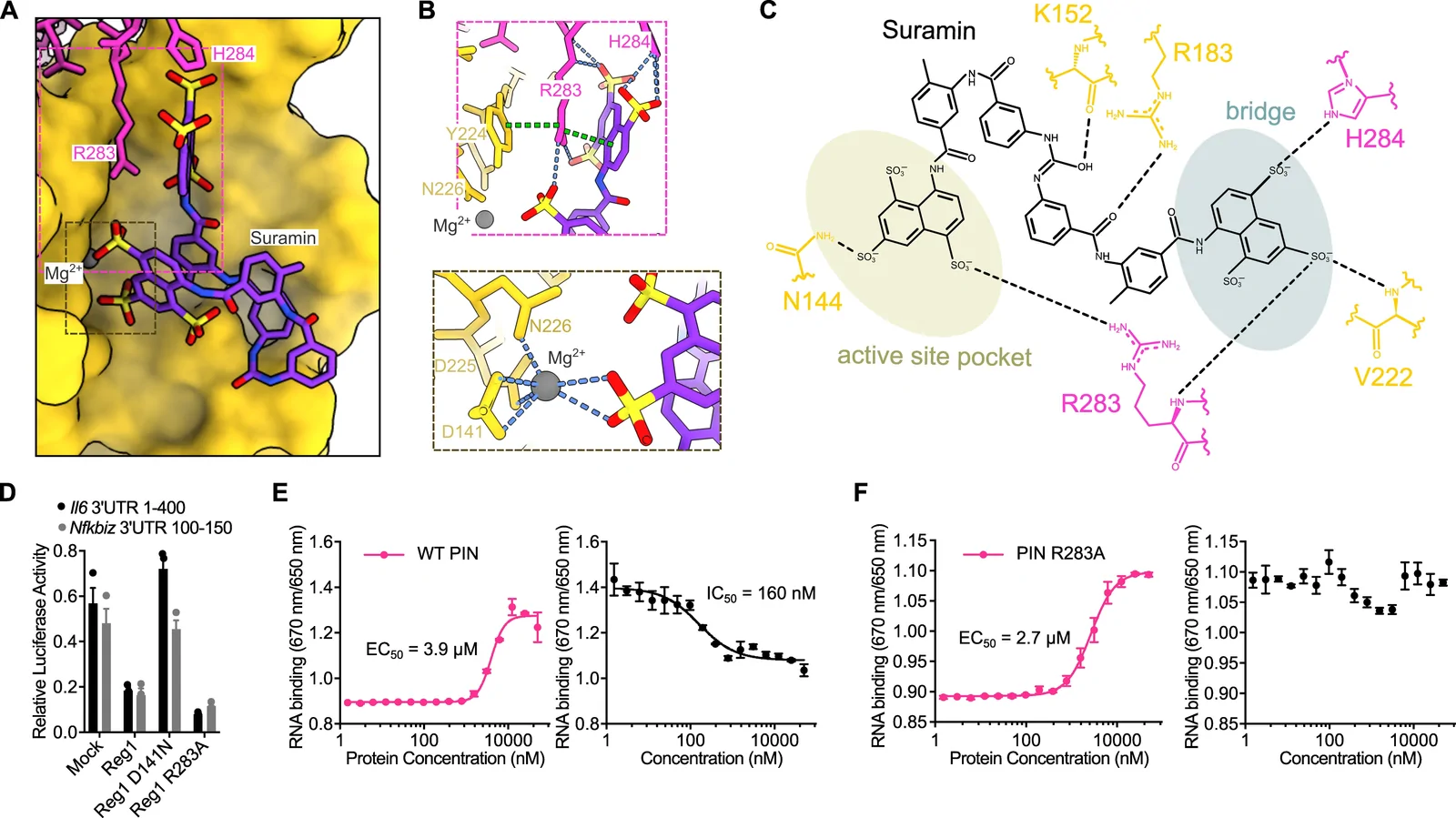
Close views of suramin binding and key interaction residues. (**A**) Close view of suramin bound at the active site of a PIN domain. (**B**) A close view of residues R283 and H284 from the adjacent PIN domain interact with suramin through hydrogen bonds (blue dashed lines) and with R283 through π–π stacking (green dashed lines). A Mg^2+^ ion interacts with several active site residues and suramin sulfonate ions. (**C**) Hydrogen bond interactions between suramin and amino acid residues in Regnase-1 PIN monomers. (**D**) Luciferase assay of the indicated Regnase-1 full-length plasmids to *Il6* 3′UTR 1–400 (black) and *Nfkbiz* 3′UTR 100–150 (gray) reporter plasmids (*n* = 3). (**E**) Binding of Cy5-labeled RNA to wild-type PIN (left) and inhibition by suramin (right). (**F**) Binding of Cy5-labeled RNA to PIN R283A (left) and inhibition by suramin (right). Error bars are s.e.m.

To evaluate the energy of interactions formed between suramin and the surrounding amino acid residues in the crystal structure and to identify their types, FMO calculations [24] were performed at the MP2/6–31G* level [29, 53]. Regnase-1 was analyzed at each amino acid residue, while suramin was divided into four fragments (f1–f4) (Supplementary Fig. S6A and B). The f1 fragment, which coordinates the Mg^2+^ ion in the active site pocket, exhibited strong and stable interactions with N144 and R283, with inter-fragment interaction energies (IFIEs) of −43.8 and −185.0 kcal/mol, respectively (Fig. 6C, and Supplementary Fig. S6C and G). Pair interaction energy decomposition analysis (PIEDA) [26] revealed that these energies are dominated by electrostatic (ES) and charge-transfer (CT) interactions, indicating the formation of stable hydrogen bonds (ES: −47.0 and −196.6 kcal/mol; CT: −5.5 and −9.3 kcal/mol, respectively). Notably, the f1 and f3 were mutually stabilized by a π–π interaction, as reflected in a large stabilizing dispersion interaction (DI) contribution of −24.8 kcal/mol (Supplementary Fig. S6C, E, and G). The f2 showed no notable interactions with Regnase-1 (Supplementary Fig. S6D). The f3 formed hydrogen bonds with the backbone of E153 and the side chain of R183, supported by large ES and CT contributions (Supplementary Fig. S6E and G). The f4 fragment formed a bridge between the two Regnase-1 PIN monomers and interacted most strongly with R283 (Fig. 6C). In addition to a predominant electrostatic interaction between the positive charge on the R283 side chain and the negative charge on the ligand f4, a cation–π or π–π interaction was observed between the guanidinium group on R283 and the naphthalene skeleton of the ligand f4 (ES: −229.6 and DI: −18.4 kcal/mol). The f4 fragment formed hydrogen bonds with the backbone NH groups of V222 and R283 and the side chain of H284, interactions supported by large ES and CT contributions (Supplementary Fig. S6F and G). These analyses provide a quantum chemical framework for interpreting the X-ray structure and identify R283 as a key determinant of suramin binding, in addition to interactions with surrounding residues. Because our FMO calculations lack a solvation model, the binding free energies should be considered as qualitative indicators of the interaction type and location, not as quantitative energetics. To validate this model, we generated an R283A mutant and analyzed its activity *in vitro* and in cells (Fig. 6D–F). PIN R283A bound RNA comparably to wild-type PIN (Fig. 6E and F), and full-length Regnase-1 R283A cleaved reporter mRNAs in cells at levels similar to wild-type Regnase-1 (Fig. 6D). However, only wild-type PIN was susceptible to suramin inhibition, whereas R283A PIN was resistant (Fig. 6E and F), which demonstrates that residue R283 is critical for Regnase-1 binding to suramin.

### Suramin derivatives are potential Regnase-1 inhibitors

To further define structure–activity relationships, we evaluated several suramin derivatives and compounds with sulfonate moieties for inhibition of Regnase-1 (Fig. 7A and B). These derivatives exhibited varying IC_50_ values, indicating that chemical modifications of the suramin scaffold modulate potency. Guided by the Regnase-1–suramin crystal structure, we performed molecular docking of 59 353 compounds from The University of Osaka Compound Library using AutoDock Vina (version 1.2.6) [33, 34] and MOE (version 2022.02) [28] (Fig. 7C), identifying several hundred candidates with predicted inhibitory activity against Regnase-1.

**Figure 7.**
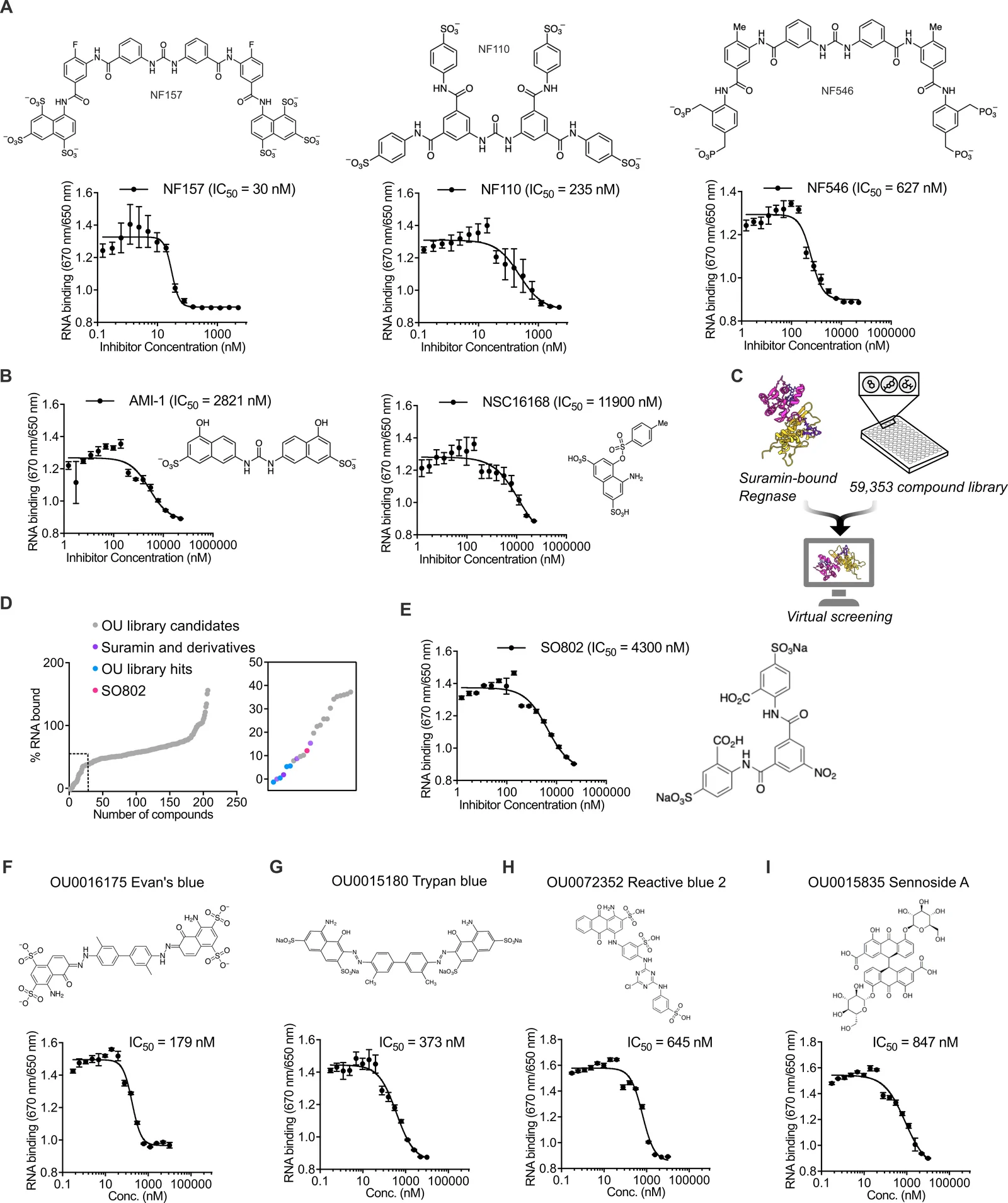
Inhibition of Regnase-1 by suramin derivatives and novel compounds. (**A**) Chemical structures of suramin derivatives NF157 (left), NF110 (middle), and NF546 (right) and the corresponding IC_50_ for the Regnase–RNA complex. (**B**) Smaller suramin-like compounds with sulfonate moieties AMI-1 (left) and NSC16168 (right) and the corresponding IC_50_. (**C**) Virtual screening and docking of compounds to the Regnase-1 PIN dimer reveals potential new compounds to inhibit Regnase. (**D**) Summary of the University of Osaka library primary screen. Normalized values of the fraction of Cy5-labeled 21-nt RNA (gray) bound to Regnase-1 NPZC D141N treated with compound versus unbound RNA. Inset shows the top 25 library candidates and suramin-like compounds. (**E**) IC_50_ of the novel compound SO802 to inhibit Regnase-RNA binding. (**F–I**) Secondary screen showing the IC_50_ of the four the University of Osaka library hits. All data are mean ± s.e.m. (*n* = 3).

Next, we defined the chemical features required for Regnase-1 binding using suramin as a structural template. Suramin is a polysulfonated naphthylurea with an approximately 2-fold-symmetric architecture centered on a urea linker and multiple terminal sulfonate groups. This highly anionic framework promotes electrostatic engagement with clusters of positively charged residues on protein surfaces. Consistent with this property, the PIN domain–suramin crystal structure showed that terminal sulfonate groups coordinate the catalytic Mg²⁺ ion while simultaneously interacting with basic residues surrounding the active site. Together, these findings indicate that the Regnase-1 catalytic pocket forms a strongly electropositive environment capable of accommodating polyanionic ligands.

A similar structure–activity relationship was observed for the suramin analogues NF157, NF110, and NF546, in which inhibitory potency depended on the number and spatial arrangement of sulfonate substituents [54, 55]. Although these compounds share a polyanionic aromatic scaffold, variation in charge spacing modulated activity, supporting a recognition mechanism driven by polyanion–cationic patch interactions analogous to engagement of the negatively charged RNA phosphate backbone. Accordingly, suramin and its derivatives function as competitive RNA mimetics despite lacking nucleotide structure.

On the basis of these structural and electrostatic principles, we designed and synthesized a suramin-inspired scaffold SO802 that captures the essential features required for Regnase-1 binding (Fig. 7E, and Supplementary Figs S7  and S8) [56].

We evaluated compounds from the University of Osaka Compound Library, the newly synthesized scaffold, suramin, and suramin derivatives using a spectral shift assay (Fig. 7D). Our scaffold SO802 inhibited Regnase-1 NPZC D141N with an IC_50_ of 4.3 µM (Fig. 7E), and several compounds from the University of Osaka Compound Library disrupted Regnase-1–RNA binding with submicromolar potency (Fig. 7F–I). Notably, most University of Osaka Compound Library hits contained sulfonate groups, supporting the importance of polyanionic functionality for Regnase-1 inhibition. One exception, sennoside A, lacked sulfonate moieties but retained a large, symmetric, hydrophilic architecture reminiscent of suramin (Fig. 7I). Together, these results further define the structural determinants of Regnase-1 inhibition and provide a framework for future inhibitor optimization. Today, suramin is still being used to treat African sleeping sickness, and researchers are still finding new roles for this century-old drug [57]. Given the exciting potential of Regnase-1 genetic knockout in CAR-T therapies [58], we hope further optimization, derivatization, and delivery of Regnase-1 inhibitors can provide additional modalities to kill cancer.

## Supplementary Material

ugag043_Supplemental_Files

## Data Availability

The PDB DOI for 23KX is https://doi.org/10.2210/pdb23kx/pdb. The data underlying the figures have been deposited to https://doi.org/10.5281/zenodo.22949290.
