# Supplementary material for: Identification of small-molecule inhibitors of the immune system repressor Regnase-1
Source: NAR Mol Med. 2026 Oct 1;3(4):ugag043. doi: 10.1093/narmme/ugag043 (PMC13684271; doi:10.1093/narmme/ugag043)
Supplement: Supplementary file 1 [file ugag043_supplemental_files.zip › 20261016_Supplement.pdf]

Supplemental Information

Identification of small-molecule inhibitors of the immune system repressor Regnase-1

Arthur Millius[1,2,3], Chee-Hong Takahiro Yew[1], Yuko Misumi[4], Jian Yu[4], Daisuke Takaya[5], Shuhei Miyakawa[5], Eun-Sol Choi[6], Hyo-Jun Lee[6], Tatum Melati Andini[1], Fahad Khalaf[1], Kazuhiko Maeda[3,7], Kaori Fukuzawa[5], Genji Kurisu[4], Shizuo Akira[3,7,8], Soyoung Park[2], and Daron M. Standley[1,2]

1) Department of Genome Informatics, Research Institute for Microbial Diseases (RIMD), The University of Osaka
2) Laboratory for Systems Immunology, Immunology Frontier Research Center (WPI-iFReC), The University of Osaka
3) Laboratory of Host Defense, WPI-iFReC, The University of Osaka
4) Protein Crystallography Laboratory, Institute for Protein Research, The University of Osaka
5) Graduate School of Pharmaceutical Sciences, The University of Osaka
6) Department of Chemistry, Kunsan National University, Gunsan, Republic of Korea
7) Department of Host Defense, RIMD, The University of Osaka
8) Center for Advanced Modalities and Drug Delivery System (CAMaD), The University of Osaka

The following information is supplemental and cannot be contained in the main body of the paper.

Supplementary Tables

Supplementary Table 1 RNAs used in this study

RNA	Name	Sequence (5'-3')	Species, Type	References
1	*21-nt synthetic RNA*	AAUGCGACAGUCGGUAGCAUC	Artificial, SELEX	refs[36]
2	*Il6* 3'UTR 84-103	GUUGUUCUCUACGAAGAACU	Mouse mRNA	refs[6]
3	*Nfkbiz* 3'UTR 100-118	GUUGUUUCUAUGAAACAAA	Mouse, mRNA	refs[52]
4	*Regnase-1* 3'UTR 193-213	GUUGAUACACAUUGUAUCUCU	Mouse, mRNA	refs[8]

Supplementary Table 2 Data collection and refinement statistics for PIN-suramin (PDB: 23KX)

**Data collection**	
Beamline	SPring-8 BL44XU
Wavelength (Å)	0.9
Resolution Range (Å)	45.92 – 2.62 (2.78 – 2.52)[a]
Space group	$P3_221$
Unit cell (a b c, α β γ)	114.72 114.72 103.56, 90 90 120
Total reflections	245692 (40427)[a]
Unique reflections	45798 (7291)[a]
Redundancy	5.4 (5.5)[a]
Completeness (%)	99.7 (98.5)[a]
Mean(I)/Sigma(I)	10.2 (1.39)[a]
Wilson B-factor (Å^2)	64.91
R-meas	0.223 (0.321)[a]
CC1/2	0.996 (0.498)[a]
**Refinement**	
Reflections used in refinement	24053 (1422)[a]
Reflections used for R-free	1211 (76)[a]
R-work	0.221 (0.457)[a]
R-free	0.264 (0.466)[a]
Number of non-hydrogen atoms	2883
Macromolecules	2699

Ligands	174
Solvent	10
Protein residues	328
RMS (bonds, Å)	0.008
RMS (angles, °)	1.38
Ramachandran	
Favored (%)	94.44
Allowed (%)	5.25
Outliers (%)	0.31
Average B-factor ($Å^2$)	75.35

a, statistics for highest resolution shell shown in parentheses

Supplementary Table 3 Interactions between suramin and Regnase-1 in the PIN-suramin structure

Suramin	Interaction	Distance (Å)	Residue (Chain)
Sulfonate 1	Hydrogen bond	2.78	Arg283 (B)
Sulfonate 3	Hydrogen bond	1.97	Arg283 (B)
	Hydrogen bond	1.78	Val222 (A)
	Hydrogen bond	2.42	Asn144 (A)
	Hydrogen bond	2.52	Asn144 (A)
	Hydrogen bond	2.78	Asn244 (A)
	Mg–O interaction	3.36	
Sulfonate 5	Hydrogen bond	1.94	Arg283 (B)
	Hydrogen bond	2.84	Arg283 (B)
	Hydrogen bond	2.71	His284 (B)
	Hydrogen bond	2.72	His284 (B)
Naphthalene ring	Cation-p-stacking		Arg283 (B)
C=O (O) of the C–D linker	Hydrogen bond	1.88	Arg283 (B)

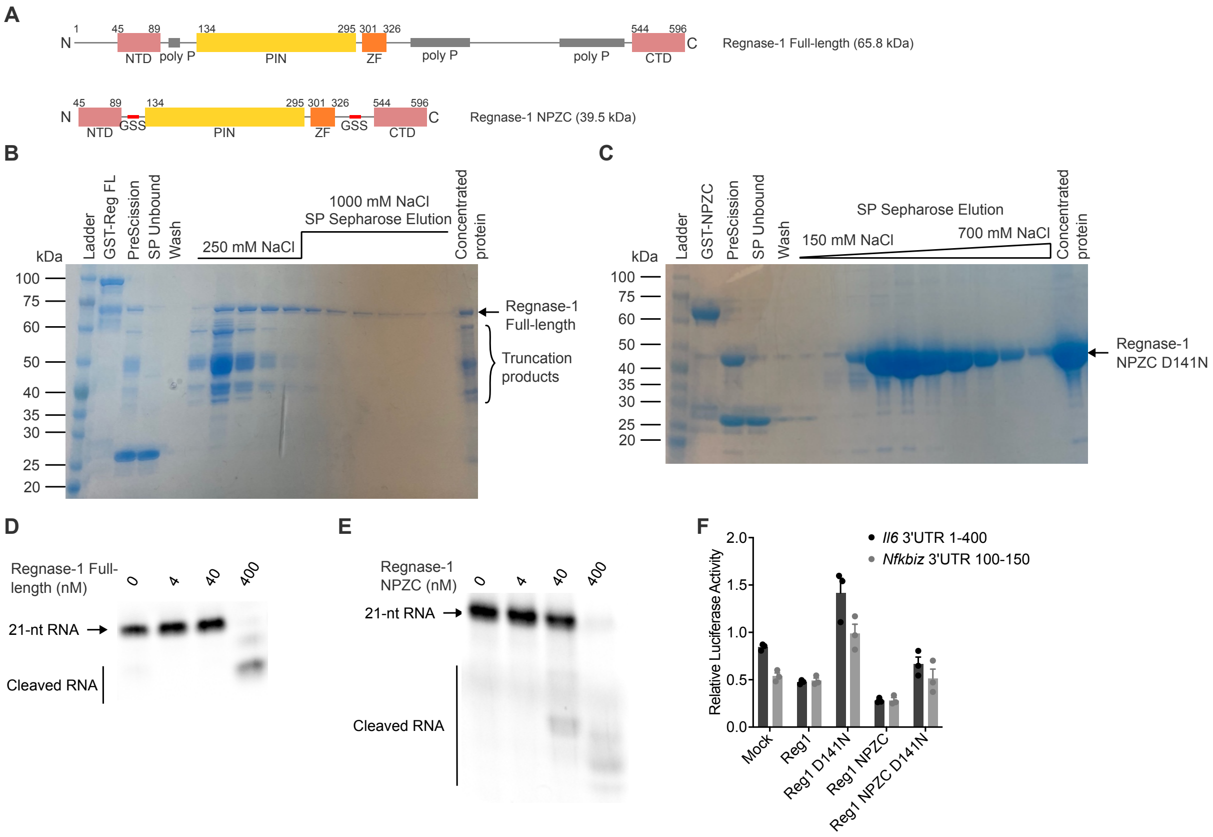

Figure S1. Purification of Regnase-1 N-terminal-PIN-Zinc Finger-C-terminal (NPZC). (**A**) Domain architecture of *Mus musculus* Regnase-1 full-length and Regnase-1 NPZC. (**B**) SDS-PAGE fractions of Regnase-1 full-length and (**C**) Regnase-1 NPZC D141N purifications. The concentrated protein is shown in the rightmost lanes. (**D**) In vitro cleavage assay of codon-optimized full-length Regnase-1 and (**E**) Regnase-1 NPZC to 21-nt synthetic RNA. (**F**) Luciferase assay of the indicated Regnase-1 full-length expression plasmids to *Il6* 3'UTR 1-400 (black) and *Nfkbiz* 3'UTR 100-150 (gray) reporter plasmids. Values are normalized to a *Renilla* control plasmid relative to an empty reporter plasmid (n = 3). Error bars are s.e.m.

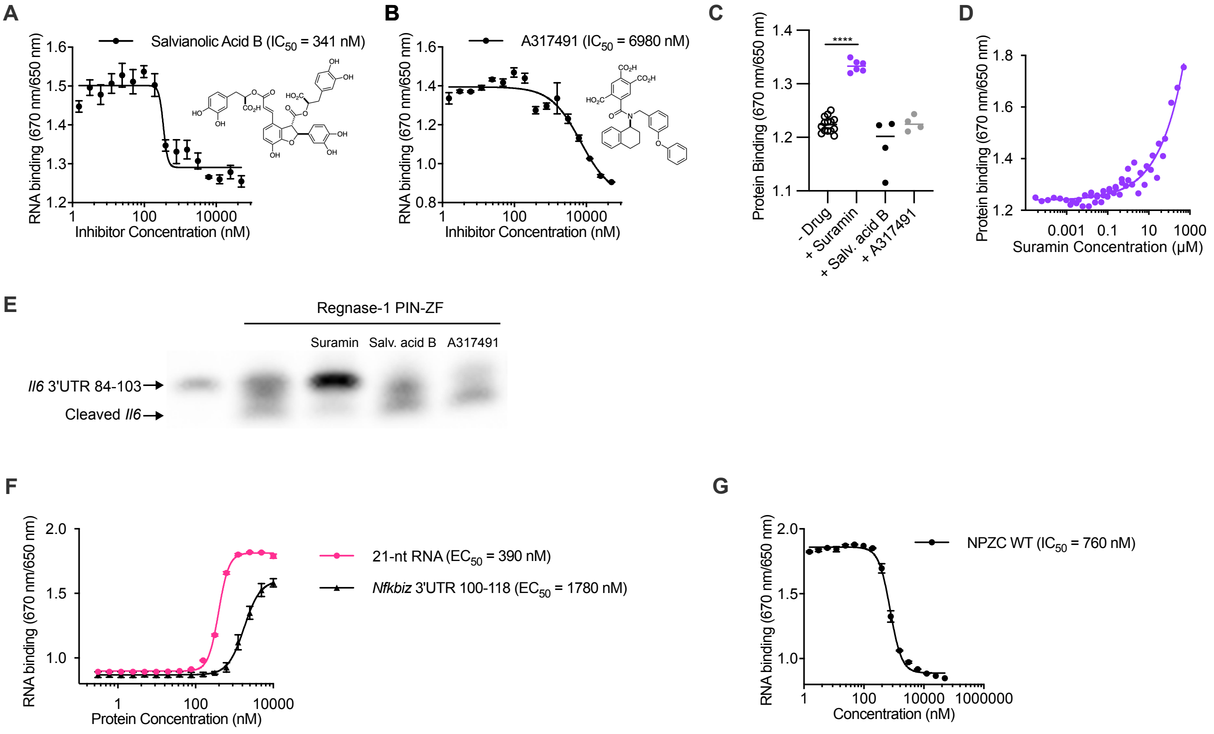

Figure S2. Suramin, but not other inhibitors, binds and inhibits Regnase-1. (**A**) IC_{50} of salvianolic acid B and (**B**) A317491 for the Regnase-1-RNA complex (n = 3). (**C**) Binding of Cy5-labeled Regnase-1 NPZC D141N to suramin (purple), salvianolic acid B (black), or A317491 (gray) versus labeled protein alone (black circles, n ≥ 4). (**D**) Binding assay of Cy5-labeled Regnase-1 NPZC to suramin. (**E**) In vitro cleavage assay of Regnase-1 PIN-ZF to Cy5-labeled *Il6* 3'UTR 84-103 in the presence of different inhibitors. (**F**) Affinity assays of 21-nt RNA (pink, n = 4) and *Nfkbiz* 3'UTR 100-118 (black, n=2) with wild-type Regnase-1 NPZC. (**G**) IC_{50} of suramin for the wild-type Regnase-1-21-nt RNA complex (n = 3). Data are mean ± s.e.m.

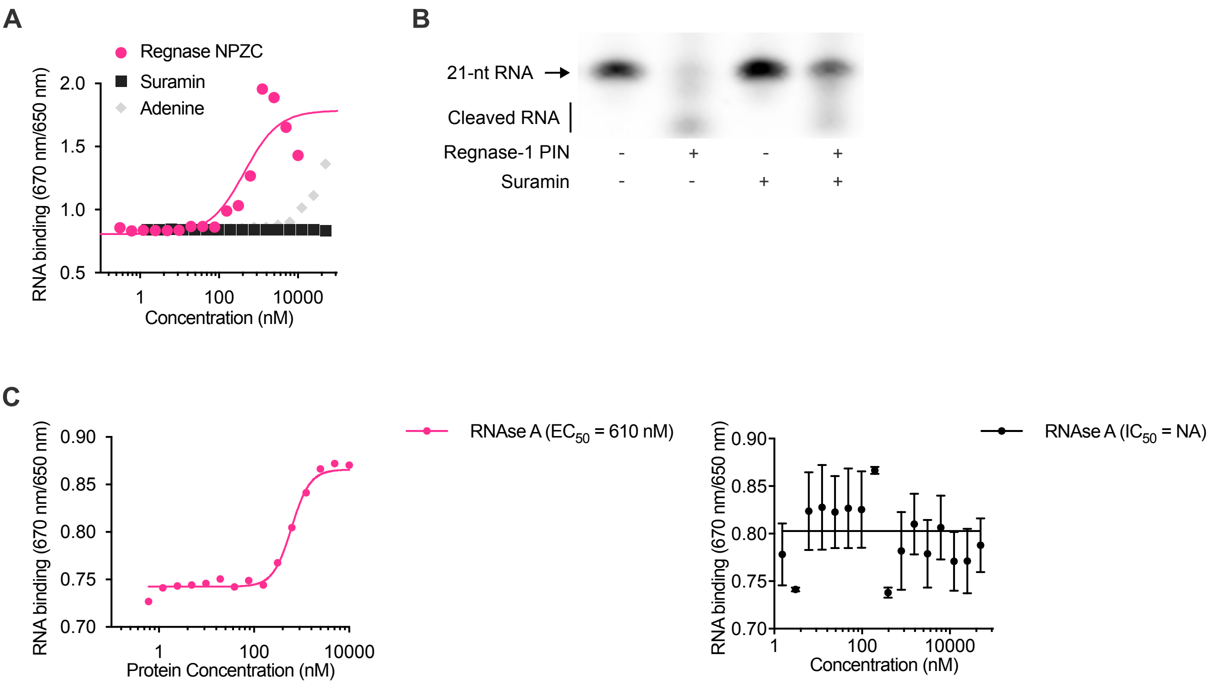

Figure S3. Suramin does not bind or degrade RNA, nor does suramin inhibit ribonucleases indiscriminately. (**A**) Affinity assays show the 670 nm/650 nm ratio for unbound Cy5-labeled 21-nt RNA to Regnase-1 NPZC D141N (pink), suramin (black), and adenine (gray). (**B**) In vitro cleavage assay of Regnase-1 PIN to Cy5-labeled 21-nt RNA with or without suramin. (**C**) Left, affinity assay of RNAse A for 21-nt RNA (n = 1). Right, IC_{50} of suramin for RNAse A-21-nt RNA complex (n = 3, mean ± s.e.m.).

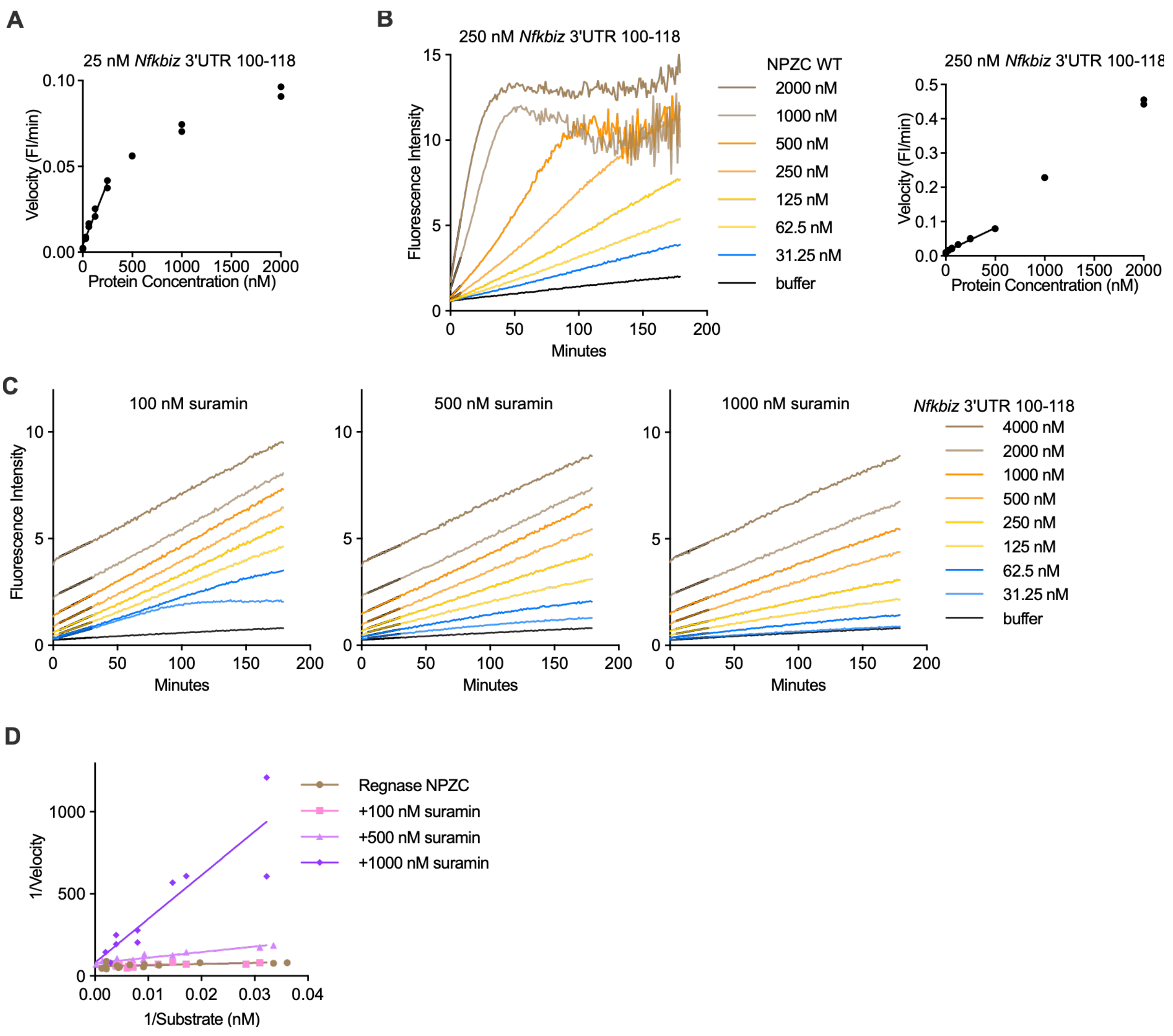

Figure S4. Validation of *Nfkbiz* substrate and underlying data for Michaelis-Menten study. (**A**) A plot of protein concentration versus reaction rate (velocity) from the data in Fig. 3B. Velocities (fluorescence intensity/min) were calculated using linear regression over the initial linear part of the reaction curve from Fig. 3B. Doubling the protein concentration doubles the reaction velocities at concentrations below 500 nM (black line, linear regression). (**B**) The experiments in Fig. 3B were repeated with a 10-fold increase in *Nfkbiz* 3'UTR 100-118 RNA substrate. The reaction reaches saturation at higher protein concentrations. Regnase-1 NPZC protein concentrations below 1000 nM show a linear increase in reaction velocity (right). (**C**) With Fig. 3C, underlying data used to create Fig. 3D. The linear part of the reaction curve was fit with a linear regression (black lines) to obtain the slope, which was adopted to reflect the reaction rate (velocity). (**D**) Lineweaver-Burk plot of the data in Fig. 3D. Linear regression (solid lines) show a similar y-intercept and increased slope for higher concentration of suramin. Data are n = 2.

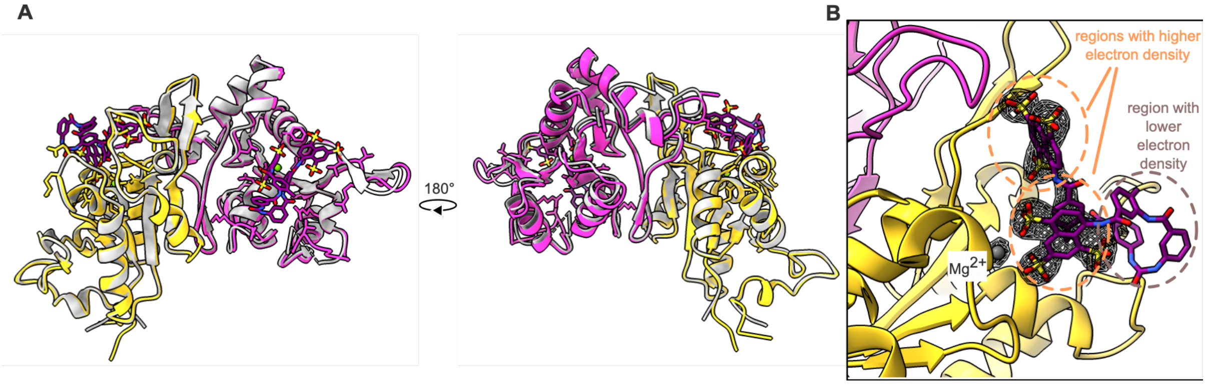

Figure S5. Structural comparison of the PIN-suramin complex with apo-PIN. (**A**) Overall view of the PIN-suramin structure (PDB ID: 23KX, yellow and magenta) overlapped with the apo-PIN structure (gray, PDB ID: 3v33). (**B**) Close-up view of suramin and Fo-Fc electron density map (mesh) at σ = 2.3 in the PIN-suramin complex.

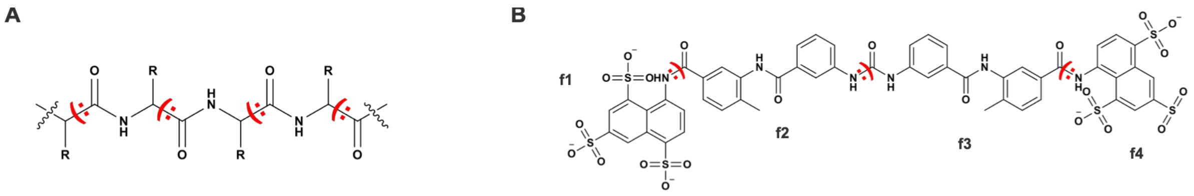

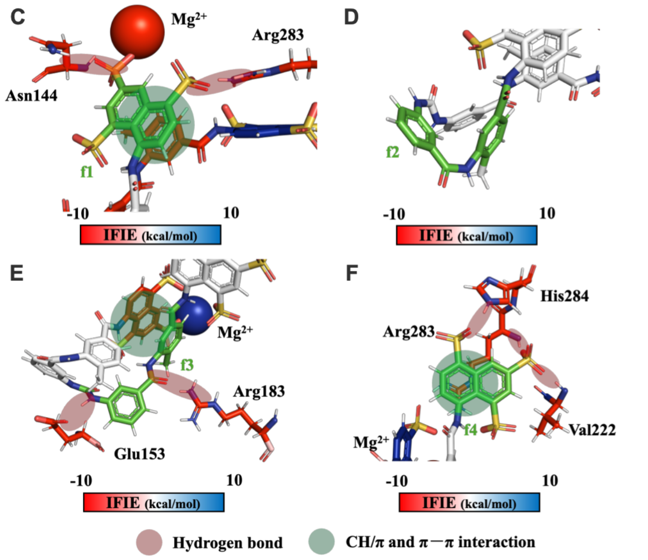

G

f1	IFIE	ES	EX	CT	DI
Asn144 (A)	-43.8	-47.0	15.9	-5.5	-7.2
Arg283 (B)	-185.0	-196.6	28.3	-9.3	-7.4
f3	-53.1	-43.2	26.5	-11.6	-24.8
**f3**	**IFIE**	**ES**	**EX**	**CT**	**DI**
Glu153 (A)	-41.2	-38.5	12.5	-8.2	-7.0
Arg183 (A)	-40.6	-41.5	13.0	-5.9	-6.3
f1	-53.1	-43.2	26.5	-11.6	-24.8
**f4**	**IFIE**	**ES**	**EX**	**CT**	**DI**
Val222 (A)	-54.0	-55.2	16.3	-6.2	-8.8
Arg283 (B)	-231.6	-229.6	25.7	-9.3	-18.4
His284 (B)	-55.8	-56.6	12.9	-5.2	-6.7

Figure S6. Fragmentation Scheme and Interaction Energy Analysis in FMO Calculations. (**A, B**) Schematic representations of the fragmentation schemes for Regnase-1 and suramin. Regnase-1 was fragmented at the amino acid residue unit, while suramin was divided into four fragments (f1–f4), numbered sequentially from the Mg^{2+}-coordinated fragment. (**C-F**) Mapping of the inter-fragment interaction energies (IFIEs) between each suramin fragment (f1–f4) and Regnase-1 onto the structure. Amino acid residues displayed as sticks are those forming significant interactions with f1–f4, defined by the criteria of |IFIE|≥5 kcal/mol, CT ≤-3 kcal/mol, or DI ≤-3 kcal/mol. Stick colors indicate stabilizing (red) and destabilizing (blue) interactions. Hydrogen bonds and CH/π or π–π interactions are shown by light red and light green ellipses, respectively. (**G**) Inter-fragment interaction energies (IFIEs) and corresponding PIEDA components for amino acid residues that form significant interactions with suramin fragments f1–f4. Significant interactions were defined by the criteria |IFIE|≥5 kcal/mol, CT ≤-3 kcal/mol, or DI ≤-3 kcal/mol.

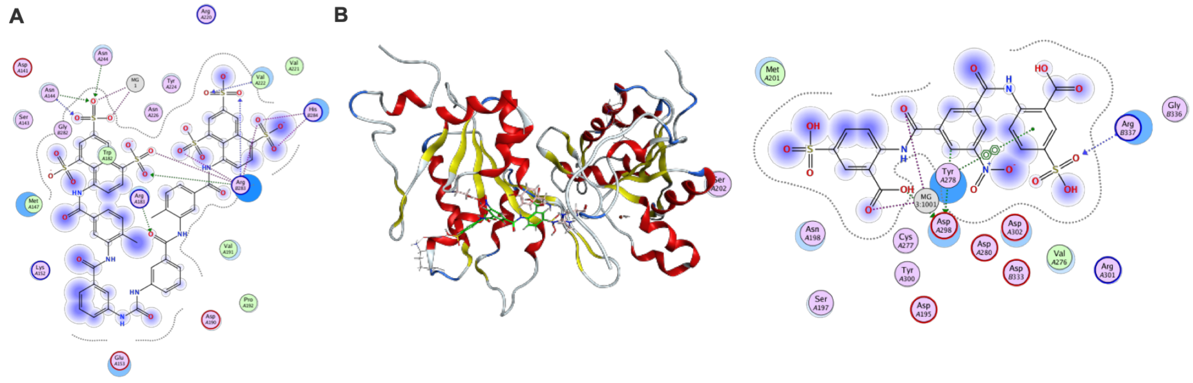

Figure S7. Coordination diagram of Regnase-1 bound by suramin and molecular docking to SO802. (**A**) Overall coordination diagram of suramin bound to PIN. Charged residues are shown as purple circles and hydrophobic residues are shown as green circles. (**B**) Molecular docking of SO802 to PIN (left) and the corresponding coordination diagram (right).

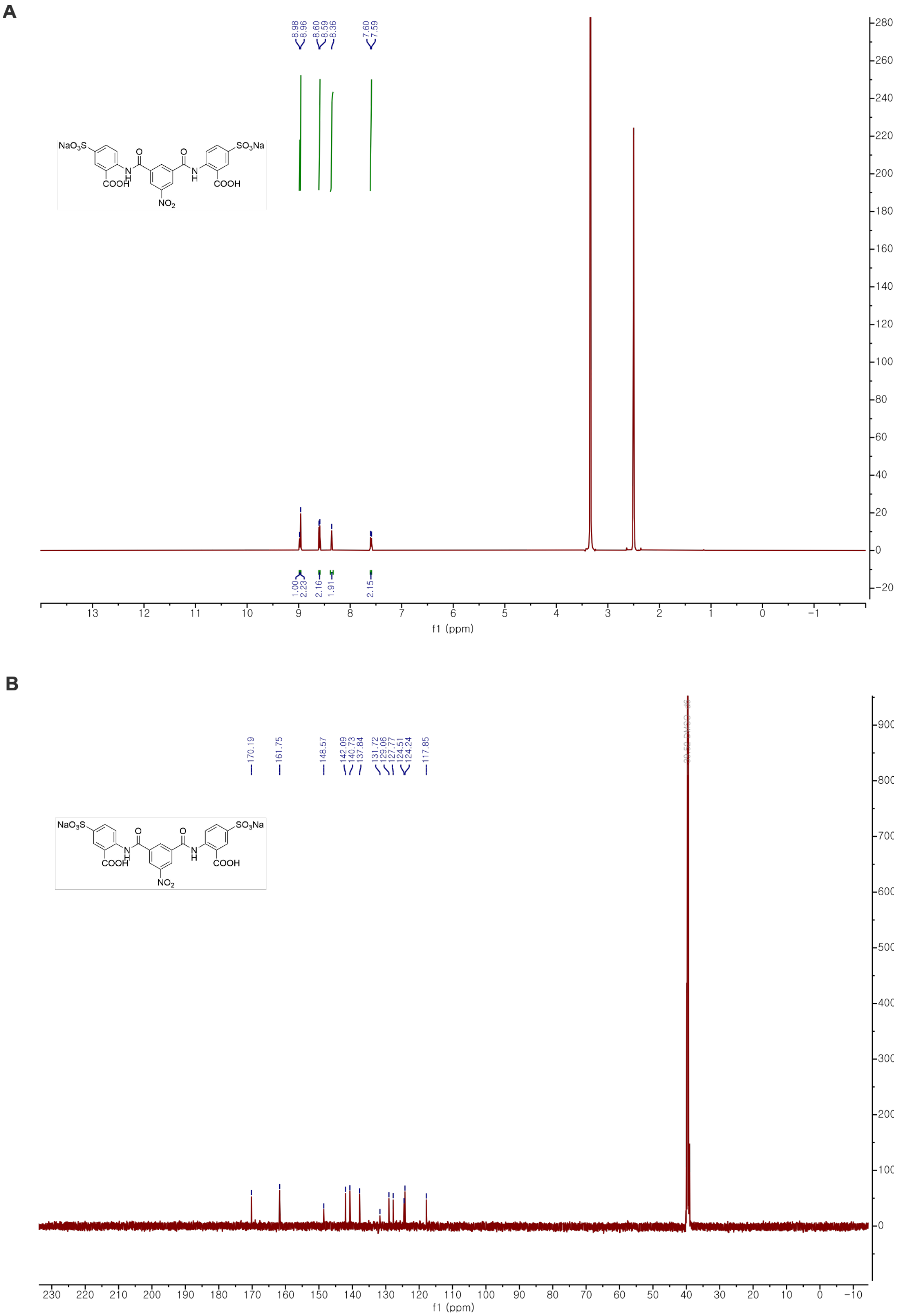

Figure S8. NMR analysis of SO802. (**A**) 1H NMR (500 MHz, DMSO) δ 8.98 (s, 1H), 8.96 (s, 2H), 8.60 (d, J = 8.5 Hz, 2H), 8.36 (s, 2H), 7.60 (d, J = 8.5 Hz, 2H) of sodium 4,4'-((5-nitroisophthaloyl)bis(azanediyl))bis(3-carboxybenzenesulfonate), which is designated SO802. (**B**) 13C NMR (125 MHz, DMSO) δ 170.1, 161.7, 148.5, 142.0, 140.7, 137.8, 131.7, 129.0, 127.7, 124.5, 124.2, 117.8 of SO802.
